# When roads appear jaguars decline: Increased access to an Amazonian wilderness area reduces potential for jaguar conservation

**DOI:** 10.1371/journal.pone.0189740

**Published:** 2018-01-03

**Authors:** Santiago Espinosa, Gerardo Celis, Lyn C. Branch

**Affiliations:** 1 Facultad de Ciencias, Universidad Autónoma de San Luis Potosí, San Luis Potosí, San Luis Potosí, México; 2 Escuela de Ciencias Biológicas, Pontificia Universidad Católica del Ecuador, Quito, Pichincha, Ecuador; 3 Center for Ecosystem Science and Society, Northern Arizona University, Flagstaff, Arizona, United States of America; 4 Department of Wildlife Ecology and Conservation, University of Florida, Gainesville, Florida, United States of America; Universita degli Studi di Sassari, ITALY

## Abstract

Roads are a main threat to biodiversity conservation in the Amazon, in part, because roads increase access for hunters. We examine how increased landscape access by hunters may lead to cascading effects that influence the prey community and abundance of the jaguar (*Panthera onca*), the top Amazonian terrestrial predator. Understanding such ecological effects originating from anthropogenic actions is essential for conservation and management of wildlife populations in areas undergoing infrastructure development. Our study was conducted in Yasuní Biosphere Reserve, the protected area with highest potential for jaguar conservation in Ecuador, and an area both threatened by road development and inhabited by indigenous groups dependent upon bushmeat. We surveyed prey and jaguar abundance with camera traps in four sites that differed in accessibility to hunters and used site occupancy and spatially explicit capture-recapture analyses to evaluate prey occurrence and estimate jaguar density, respectively. Higher landscape accessibility to hunters was linked with lower occurrence and biomass of game, particularly white-lipped peccary (*Tayassu pecari*) and collared peccary (*Pecari tajacu*), the primary game for hunters and prey for jaguars. Jaguar density was up to 18 times higher in the most remote site compared to the most accessible site. Our results provide a strong case for the need to: 1) consider conservation of large carnivores and other wildlife in policies about road construction in protected areas, 2) coordinate conservation initiatives with local governments so that development activities do not conflict with conservation objectives, and 3) promote development of community-based strategies for wildlife management that account for the needs of large carnivores.

## Introduction

Many populations of large carnivores are declining and threatened with extinction worldwide [1]. Because large predators have been shown to play key roles in the structure and function of many ecosystems, reduction in populations of these species has altered dynamics of ecosystems ranging from grasslands to tropical forest [1,2]. Major causes of large carnivore declines include habitat loss and degradation with human development, mortality from human-carnivore conflicts or poaching, prey depletion, and synergisms among these factors [1,3,4]. As a result of these processes, in the last century, the jaguar (*Panthera onca*) has disappeared from over half of its original range [5,6].

Currently, the Amazon Basin is the main stronghold for jaguar conservation as it sustains 89% of the cat’s global population [6]. However, Amazonia faces rapid changes associated with large-scale development that can threaten the future of the jaguar in the region. For example: 80,000 km^2^ of forests have been converted to soybean plantations in the Brazilian Amazon [7]; hydropower dams exist, are under construction, or planned in numerous tributaries of the Amazon [8]; and extractive activities, such as those for hydrocarbons and minerals, are widespread in the region and in some cases occur within protected areas [9,10]. These developmental activities are associated with road development that promotes formation of new settlements, increases colonization rates, and ultimately catalyzes land cover change and biodiversity loss [11–13].

In face of current and future change in Amazonian ecosystems, jaguar conservation depends heavily upon the large system of protected areas, particularly megareserves, in this region (i.e., areas > 10,000 km^2^, [14,15]. However, pressure to construct roads within wilderness areas is high [9,16]. Traditionally, access by hunters has been limited to areas adjacent to rivers. As new roads are developed in the region, we can expect that a higher proportion of Amazonia will become accessible, and therefore the proportion of natural areas that function as wildlife refuges or sources will decrease [17–19]. Moreover, as road networks increase, markets become more available to subsistence hunters promoting commercialization of wildlife and targeted hunting of large game species that provide high returns [19–23]. The consequences of this process for jaguar populations may be large because programs for sustainably managing hunting generally are lacking, and density of large carnivore populations often is closely related to abundance of large-bodied prey, such as ungulates, which increasingly are overexploited [24–26].

Based on these observations, one can infer that the potential of protected areas to conserve large carnivores such as jaguars may be seriously compromised if these lands become more accessible by roads. Thus far, studies that contribute to understanding cascading effects of roads on wildlife in tropical forests have primarily investigated impacts of roads on hunting pressure [e.g., 18,20,22]. In this paper we analyze the consequences of increasing accessibility to a wilderness area for populations of jaguar and their prey. This research was conducted in Yasuní Biosphere Reserve located in Ecuador’s Amazon region where pressure to extract natural resources is high [27–29].

## Materials and methods

### Study area

**Fig 1:**
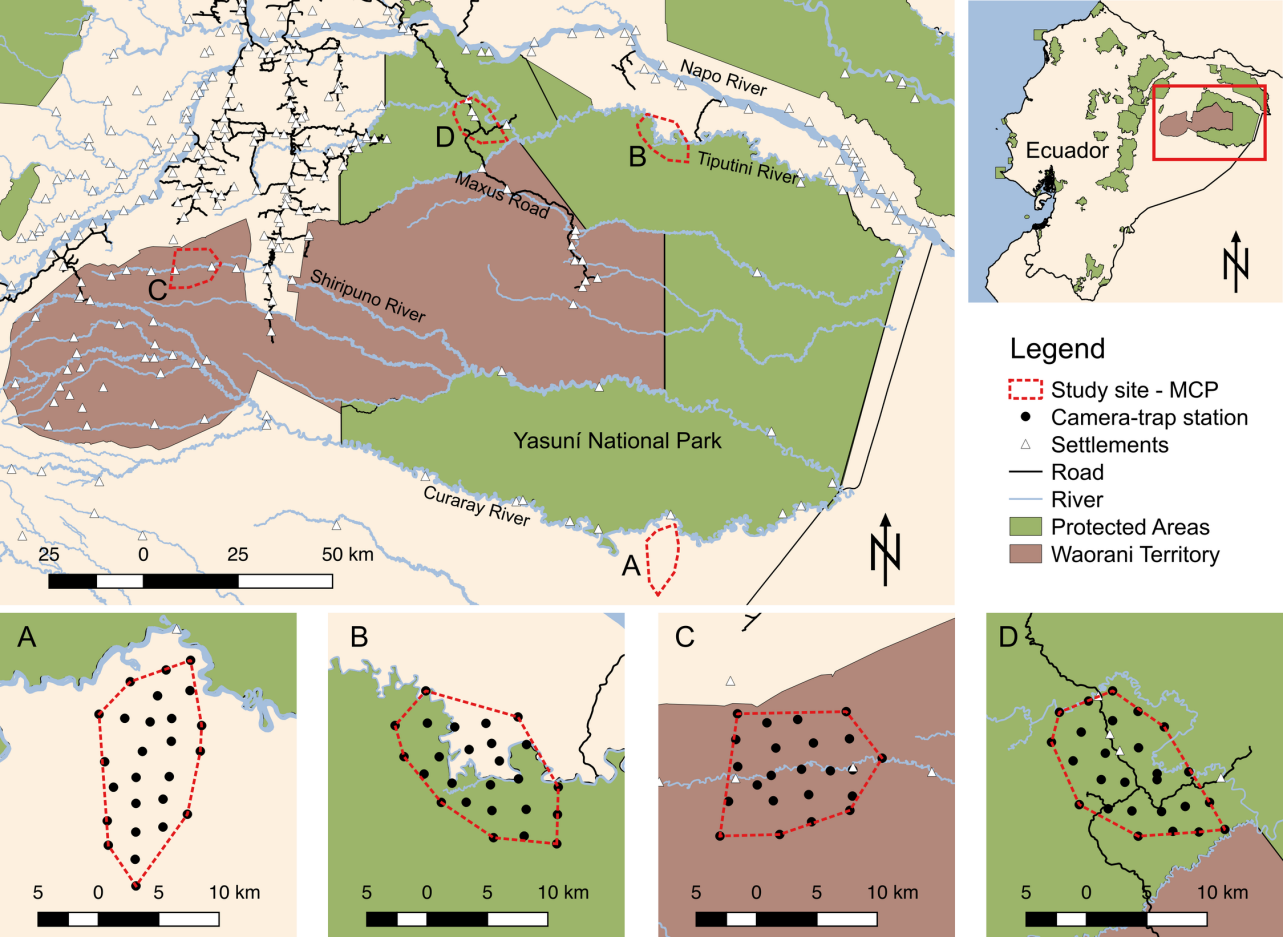
Camera trap arrays in four sites in Yasuní Biosphere Reserve ordered from low to high human access. A) Lorocachi; B) Tiputini; C) Keweriono; D) Maxus Road.

Yasuní Biosphere Reserve (hereafter Yasuní) is located in the Ecuadorian Amazon and formed by Yasuní National Park (ca. 10,000 km^2^) and the adjacent Waorani Ethnic Reserve (ca. 8,000 km^2^) (Fig 1). Yasuní is mainly inhabited by the Waorani, a historically semi-nomadic group that began adopting a sedentary lifestyle after their contact with western culture in the mid-1950s [30]; other indigenous groups, such as Kichwa and Shuar, live at the reserve margins. Road construction began in the early 1980s with the Auca Road to access oil reserves in Waorani territory. In the early 1990s, the Maxus Road was built in the northern portion of Yasuní National Park (Fig 1) and various permanent Waorani settlements were formed along this road.

Vegetation in Yasuní primarily comprises evergreen tropical *terra firme* forest with canopy height between 25–40 m [31]. Flood plains and swampy areas dominated by the palm *Mauritia flexuosa* occur along margins of the main rivers. Seasons are not marked. Annual rainfall is ~3,000 mm with >100 mm precipitation in all months. Mean monthly temperatures are 22–34°C [31].

### Study design

We hypothesized that in Yasuní: 1) abundance of large game is strongly influenced by landscape accessibility to hunters, and 2) reductions of large game cause local declines in jaguar density. Indigenous inhabitants in these areas generally do not hunt jaguars [19,32–34]. Based on this, we predicted that occurrence and biomass of prey would be lower in more accessible areas and that we would observe lower local densities of jaguars in these areas. To evaluate our predictions, we surveyed prey and jaguar populations in four sites in Yasuní with varying degree of access. Ordered from the least to the most accessible, these sites were Lorocachi [76°00’30”W, 1°42’23”S], Tiputini [76°00’07”W, 0°42’35”S], Keweriono [77°07’27”W, 1°00’49”S], and Maxus Road [76°26’49”W, 0°40’11”S] (Fig 1; Table 1).

**Table 1:** Hunter’s accessibility at four study sites in Yasuní. Accessibility is measured as distance (mean km ± SD) of camera traps to three sources of access: roads, rivers and settlements.

	Study sites			
Source of access	Lorocachi	Tiputini	Keweriono	Maxus Road
Road	74.46 ± 4.70	31.72 ± 2.72	12.34 ± 2.91	1.85 ± 1.25
River	6.69 ± 1.18	1.70 ± 1.49	2.25 ± 1.67	2.28 ± 1.76
Settlement	11.61 ± 5.09	15.06 ± 2.93	2.96 ± 1.53	3.56 ± 1.62

Lorocachi (Fig 1A) is located near the Curaray River adjacent to the southern border of Yasuní in the buffer area of the reserve and is accessible only by air. This site is inhabited by a Kichwa community of 120 people and a 300-person army base, both established in 1953. Army personnel are not allowed to hunt and are provisioned by Ecuador’s army. Protein demands of the Kichwa are met with poultry, fishing, and hunting. Kichwa in Lorocachi have no-hunt areas and limited harvest of Salvin’s curassow (*Mitu salvini*), Amazonian tapir (*Tapirus terrestris*) and white-lipped peccaries (*Tayassu pecari*) within their hunting area [35]. Our survey area was accessible only on foot and extended from 3 to 21 km from the community of Lorocachi. Based on maximum distances walked by Amazonian hunters from settlements or sources of access (i.e., 8–9 km), we estimate that 70–80% of this survey area is out of reach of hunters [18,19].

The Tiputini site (Fig 1B) is located on the northern margin of Yasuní accessible only by the Tiputini River. Although this area is not inhabited, we observed ammunition, remains of hunting camps, and Waorani and Kichwa hunters while conducting our study (one and two occasions, respectively). As hunters have to travel 3–5 hours from their settlements by dugout canoes to reach this site, it is likely that hunting occurs sporadically.

The Keweriono site (Fig 1C) is within the Waorani Ethnic Reserve in the western portion of Yasuní, and includes the settlements of Apaika (10 inhabitants) and Keweriono (60 inhabitants) along the Shiripuno River. Apaika and Keweriono are 15 and 25 km from the Auca Road, respectively. All our survey area is accessible to hunters by foot, and hunting has been continuous in this area since establishment of Keweriono in 1989. Hunting is principally for subsistence purposes, but smoked bushmeat is occasionally traded along the Auca Road [30,33]. Hunters from Apaika and Keweriono can get to the road on foot (6–8 hrs) or by traveling on the river (3–6 hrs).

The Maxus Road site (Fig 1D) encompasses hunting areas of four Waorani settlements (Guiyero, Tiwe, Ganketa and Timpoka) with a combined population of 70. All this area is accessible to hunters by foot and has been hunted continuously since settlements formed in the early 1990s after the road was constructed. Bushmeat extraction is greater at this site than our other study sites as Waorani along this road use wildlife as a commodity, trading approximately 35% of the total harvest at local markets [19].

### Wildlife survey

To evaluate occurrence of prey and jaguar density across areas that differed in accessibility to hunters, we conducted semi-systematic sampling with camera traps (Leaf River™ model C1-BU equipped with passive heat and motion sensors) for 90 consecutive days at each of our four sites. We placed 23–26 camera trap stations (hereafter stations) within a polygon of 104–110 km^2^ at each site (S1 Table). Stations were located 2–3 km apart along transects cut for this study (44–67 km of transects per site) with cameras on opposite sides of the transect opening to photograph both flanks of an animal and, thus, maximize the probability of identification of individual jaguars from fur rosette patterns. Spacing of stations was designed to ensure that all jaguars within each survey area had some probability of capture [36]. Cameras were mounted on trees 30–40 cm above ground and tested to ensure capture of prey species as small as black agouti (*Dasyprocta fuliginosa*) and nine-banded armadillo (*Dasypus novemcinctus*). We placed stations in optimal microsites (e.g., where we observed animal trails and jaguar or prey tracks) and only in *terra-firme* forest to prevent equipment from flooding. Stations were baited with six drops of a mix of Hawbacker’s Wildcat lures No. 1 and 2 (S. Stanley Hawbaker & Sons, Fort Loudon, PA), which we placed on a piece of woody debris between camera traps at installation time and every 15 days when we checked cameras.

Our research protocol was reviewed by the Ministry of the Environment of Ecuador and this Ministry granted us a permit to work in Yasuní National Park (Permit No. 012-IC-FA-PNYRSO). We also obtained a permit from the Waorani Organization (NAWE) to work in their territory. Our research protocol did not include invasive techniques such as animal handling or sacrifice and was approved by the Institutional Animal Care and Use Committee (IACUC-E812) of University of Florida.

### Analyses of prey availability

Prey availability for jaguars at the four sites was estimated using two measures: 1) occurrence of prey species measured as probability of site occupancy *Ψ* [37], and 2) biomass per camera trap station. *Ψ* may reflect the chance jaguars have to encounter prey at a particular site, and prey biomass is a measure of potential food availability. Prey occurrence and detection probability were estimated with single season site occupancy models with program PRESENCE [37,38]. Because medium-sized and large mammals are highly mobile and could enter and leave the survey area during our sampling, the occupancy estimator is best interpreted as probability of site use rather than probability of occupancy [37]. We use traditional occupancy terminology in this paper for ease of presentation. We developed separate occupancy models for all species from our study area that have been reported to be important prey for jaguar: white-lipped peccary, collared peccary (*Pecari tajacu*), Amazonian tapir, red brocket (*Mazama americana*), Amazonian brown brocket (*M*. *nemorivaga*), paca (*Cuniculus paca)*, black agouti, and armadillos (*Dasypus novemcinctus* and *D*. *kappleri*; analyzed as a single species because of uncertainty in species identification from pictures) [39].

To estimate occupancy for these 8 prey species, we combined data from our four study sites (n = 100 stations) and divided the 90-day survey period into 9 trapping occasions of 10 days each (S1 Data). We modeled occupancy as a function of landscape accessibility to hunters represented by two covariates: Euclidean distances of stations to settlements and to nearest source of access—road or navigable river. Because hunting by subsistence hunters is concentrated around settlements and near margins of rivers and roads, distances to these landscape features are good predictors of hunting intensity [19,40]. We added habitat type as a third predictor of occupancy represented by a dummy variable that reflects the main topographical categories in *terra firme* in Yasuní (ridges and valleys [31], S1 Data). We used distance between paired cameras as a predictor of detection probability and developed 15 models for each species that included all combinations of explanatory variables for occupancy and detection probability. Best-fit models were selected with Akaike Information Criterion corrected for small samples (AIC_c_, [41]. We obtained site-specific *Ψ* estimates by averaging the *Ψ* estimates of stations within sites.

To assess the prey base for jaguars, we estimated a biomass index for each station defined as: $BI=\frac{\sum_{i=1}^{R}n_{i}w_{i}}{t}$, where *R* is the number of species detected in a station, *n*_*i*_ is the number of individuals of species *i*, *w*_*i*_ is the average weight (kg) of species *i*, and *t* is the time (days) the station was active. Average weight of each species was obtained from animals hunted along the Maxus Road in a parallel study [19] or from the database PanTHERIA [42]. We estimated three *BIs* per station that included: 1) all terrestrial species with body mass ≥1 kg, 2) only ungulates, and 3) all terrestrial species excluding ungulates. We excluded puma (*Puma concolor*) from these analyses because it is unlikely that jaguars prey on this large cat. Pumas have not been reported as prey in studies of jaguar diet [43–50]. To estimate *BIs* we considered photographs of the same species to be independent when a minimum of 1 hour occurred between detections. If more than one picture of a species was taken within a one-hour period, we chose the picture with the largest number of individuals for *BI*. Our *BI* is a measure of biomass per unit of time (kg/day) for each station and therefore does not strictly reflect biomass (i.e., mass of living material per unit of area), however, we use the term biomass throughout our manuscript for convenience. We tested for differences in biomass among our four study sites with a Kruskal-Wallis test.

Finally, to evaluate the relationships between ungulate prey biomass and measures of landscape accessibility (distance to road, river, and settlement) we conducted a Pearson correlation. We estimated coefficient 95% highest density intervals using Bayesian bootstrap method with 1,000 posterior resamples draws with R package ‘bayesboot’ [51]

### Estimation of jaguar density

We used two approaches to estimate jaguar density at each site based on adult individuals captured within the 90-day sample period. First, we estimated jaguar density with a spatially explicit capture-recapture model (SECR). SECR models do not rely on estimating an effective trapping area and, therefore, are particularly useful in the absence of geographic closure. We used a Bayesian approach for SECR, implemented by R package SPACECAP [52,53], which relies on Markov chain Monte Carlo (MCMC) simulation for parameter estimation and is less sensitive to small sample sizes than methods using asymptotic inference [52,54]. Density estimation with SECR involves two groups of models. A state process model simulates potential individual home range centers that are uniformly distributed within an area, the state-space *S*, which includes the trapping array. The size of *S* must be large enough that no animals captured on the trapping array have a probability of being captured outside of *S*. A second group of observation models simulates detection probability of individuals within *S*. Observation models include a detection function and a capture encounter model and provide two basic parameters: 1) *λ*_*0*_, the detection probability when the distance between an animal’s home range center and camera trap equals zero; and 2) *σ*, which represents the spatial scale at which detection probability decreases.

Bayesian-SECR models use data augmentation that consists of adding an extra number of individuals *M* with “all-zero” capture histories. *M* is assumed to contain the true population number, *N*, within *S*. In the context of Bayesian inference, *M* can be interpreted as an upper bound of an uninformative uniform prior (0, *M*) for *N* [52,53]. SPACECAP provides estimates of population size in the larger state-space (*N*_*super*_), Density (*D*_SECR_), and *ψ*, which is the fraction of *M* that actually represents the true population [53].

For each site, we ran null models that described the detection function with a half-normal distribution and capture encounters with a Bernoulli process. To define *S* we used a 15-km buffer around each trap array envelope, with potential individual home range centers distributed uniformly at a density of one per km^2^. Potential home range centers were associated with a dummy variable of habitat suitability (i.e., 1 for suitable, 0 otherwise). All data used for jaguar density estimation with SPACECAP are available in S2 Data. For data augmentation we used 20–40 times the number of individuals observed in each site (S2 Table). We ran MCMC simulations with a burn-in period of 50%, a thinning rate of 10 and increased the number of iterations progressively, from 50,000 to 600,000, until convergence of parameters was achieved (S2 Table). We evaluated model convergence with the z-statistic derived from the Geweke’s diagnostic on the Markov chain Monte Carlo analysis; values of the z-statistic between -1.6 and 1.6 indicate model convergence [53].

For our second approach we estimated density as: $D=\frac{\hat{N}}{ETA}$, where $\hat{N}$ is an estimate of population size derived from a capture-recapture model of closed populations, and *ETA* is an estimate of effective trapping area. We estimated $\hat{N}$ with program CAPTURE [55] and used the M_h_ model with a jackknife estimator to allow for different capture probabilities among individuals, which has more biological meaning than assuming capture homogeneity [56]. Estimation of $\hat{N}$ has two critical assumptions: 1) demographic closure (i.e., no migration, births or deaths) during the study period; and 2) all individuals have a probability greater than zero of being captured [57]. We developed one matrix per site where rows corresponded to *i* individuals identified at each site and columns to *j* trapping occasions. A trapping occasion was defined as three consecutive trapping days (i.e., total of 30 trapping occasions per site). The entry in the *X*_*ij*_ matrix was 1 if an individual was recorded during a trapping occasion and 0 otherwise (S2 Data).

The $\hat{N}/ETA$ method has been widely used to estimate jaguar density in other areas, despite its limitations, and thus facilitates comparisons across studies [58]. Estimates of density from $\hat{N}/ETA$ are dependent on trap spacing and the number of recaptures of individuals [59], which can be problematic when studying large felids that have large home ranges and naturally low abundance. Also, although estimation of $\hat{N}$ is straightforward, estimation of *ETA* is difficult when sampling areas have no physical boundaries. To address this problem, a boundary strip often is placed around the sampling area that equals half or the full mean maximum distance moved (MMDM) by individuals derived from captures of individuals at different traps [56,60,61]. We used the full MMDM as a buffer around each station because this buffer provides more conservative density estimates and is supported by telemetry and simulation studies of jaguar density [60,62]. To estimate MMDM we used the pooled maximum distances of all study sites and excluded recaptures of individuals at the same stations. This MMDM was 6.08 ± SE 0.73 km estimated from 15 jaguars. We calculated the standard error of density with the formula provided in Karanth and Nichols [56].

## Results

We obtained a total of 4,565 independent photographs of terrestrial mammals and birds with body size ≥1kg, corresponding to 24 species of mammals (3,454 photographs) and 5 species of birds (1,111 photographs; S3 Table).

### Prey availability

**Fig 2:**
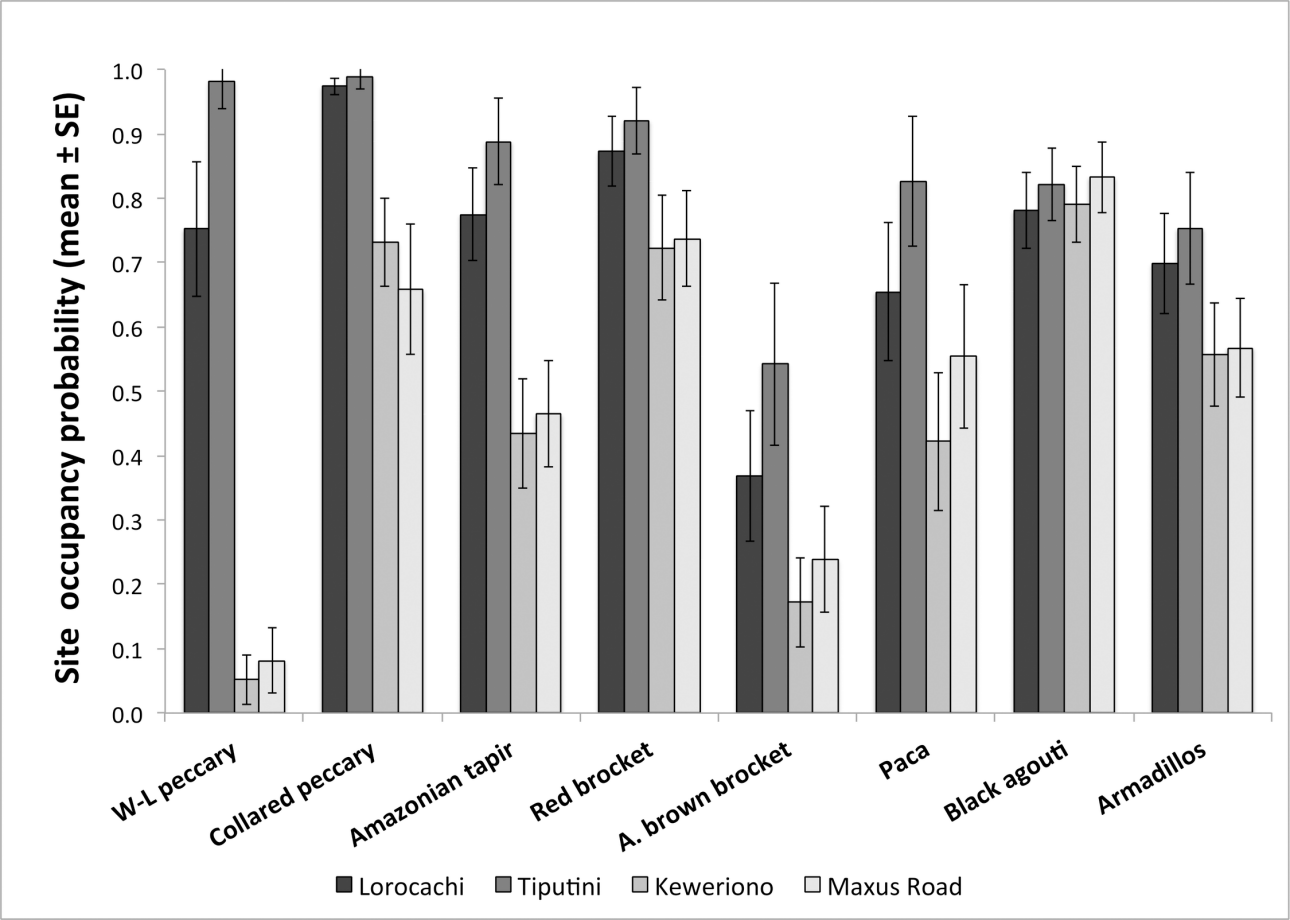
Probability of site occupancy of eight important jaguar prey species at four sites in Yasuní Biosphere Reserve. Sites are arranged from least to most accessible (dark to light). As a point of clarification, white-lipped peccaries were never recorded in camera traps at Keweriono, however, occupancy models predict a small probability of occurrence of this species at this site based on covariates.

Accessibility of the forest to humans was an important predictor of occurrence for 7 of the 8 prey species analyzed (Table 2; S1 Appendix). For all species except black agouti, best-fit models of occupancy included distance to settlements as a covariate with a positive coefficient, indicating that the further the distance from settlements the higher the occurrence of prey. Distance to roads and rivers also was an important predictor of occurrence of collared peccaries, and type of *terra firme* (ridge vs. valley) was included in best-fit models for the two large rodents and brown brocket deer (Table 2; S1 Appendix). At the least accessible sites, Lorocachi and Tiputini, occurrences of the five species of ungulates were notably higher than in the most accessible sites, Keweriono and Maxus Road (Fig 2). The most extreme case was white-lipped peccaries which were never detected in Keweriono and scarcely detected in the Maxus Road, but exhibited an occupancy probability ≥ 0.75 at other sites. Also, the occurrence of armadillos and pacas was 15–50% higher in the two least accessible sites. In contrast, occurrence of agoutis did not differ with accessibility of sites to humans (Fig 2).

**Table 2:** Occupancy models for jaguar prey in Yasuní. Untransformed estimates of coefficients for covariates *β* (SE) to predict prey probability of site occupancy *Ψ* as a function of: distance to nearest road or river (*RR*), distance to nearest settlement (*ST*) and habitat (*H*). Detection probability *p* is modeled as a function of distance between paired cameras (*DC*) or as constant (.).

Species, best-fit model	Intercept *Ψ*	*β*_*RR*_	*β*_*ST*_	*β*_*H*_	Intercept *p*	*β*_*DC*_
White-lipped peccary, *Ψ(ST)p(DC)*	-5.73	-	0.77	-	-2.78	0.19
Collared peccary, *Ψ(RR + ST)p(DC)*	-2.26	1.63	0.43	-	-1.49	0.10
Amazonian tapir, *Ψ(ST)p(DC)*	-0.88	-	0.20	-	-3.13	0.21
Red-brocket, *Ψ(ST)p(DC)*	0.59	-	0.13	-	-3.49	0.30
Amazonian brown-brocket, *Ψ(ST + H)p(*.*)*	-1.38	-	0.13	-1.03	-1.62	-
Paca, *Ψ(ST + H)p(*.*)*	0.14	-	0.14	-1.37	-1.52	-
Black agouti, *Ψ(H)p(*.*)*	1.91	-	-	-0.82	-0.52	-
Armadillos, *Ψ(ST)p(DC)*	0.01	-	0.07	-	-0.60	-0.09

Prey biomass also declined as human access increased. Overall mean prey biomass was greater by a factor of 2.0–4.5 at the two sites with lowest human access as compared to the high access sites (Kruskal-Wallis *X*^*2*^ = 52.43, df = 3, *P* < 0.001, Table 3). These differences were driven by 2.5–7.8 fold changes in the total biomass of ungulates between low access and high access sites (Kruskal-Wallis *X*^*2*^ = 55.69, df = 3, *P* < 0.001, Table 3). For example, biomass of white-lipped peccaries was 15 and 46 times higher in Lorocachi and Tiputini, respectively, than in Maxus Road. Biomass of collared peccaries was 2–3 times higher in the two most isolated sites than in the most accessible sites (S3 Table). Biomass of non-ungulate prey species was similar among all 4 sites (Kruskal-Wallis *X*^*2*^ = 0.84, df = 3, *P* = 0.839; Table 3).

**Table 3:** Biomass of jaguar prey at four sites in Yasuní Biosphere Reserve. Data are the average of biomass indexes (kg/day) for camera trap stations at each site. Lorocachi and Tiputini are the most isolated sites and Keweriono and Maxus Road are the most accessible sites.

	Study sites			
Biomass	Lorocachi (n = 26)	Tiputini (n = 25)	Keweriono (n = 23)	Maxus Road (n = 26)
All prey species	10.23 ± 7.06	16.57 ± 7.13	3.65 ±2.00	4.97 ±3.51
Ungulates	8.4 ± 6.9	14.73 ± 6.98	1.90 ± 1.85	3.28 ± 3.06
Non-ungulates	1.84 ± 1.14	1.83 ± 0.94	1.75 ± 0.88	1.69 ± 1.13

**Fig 3:**
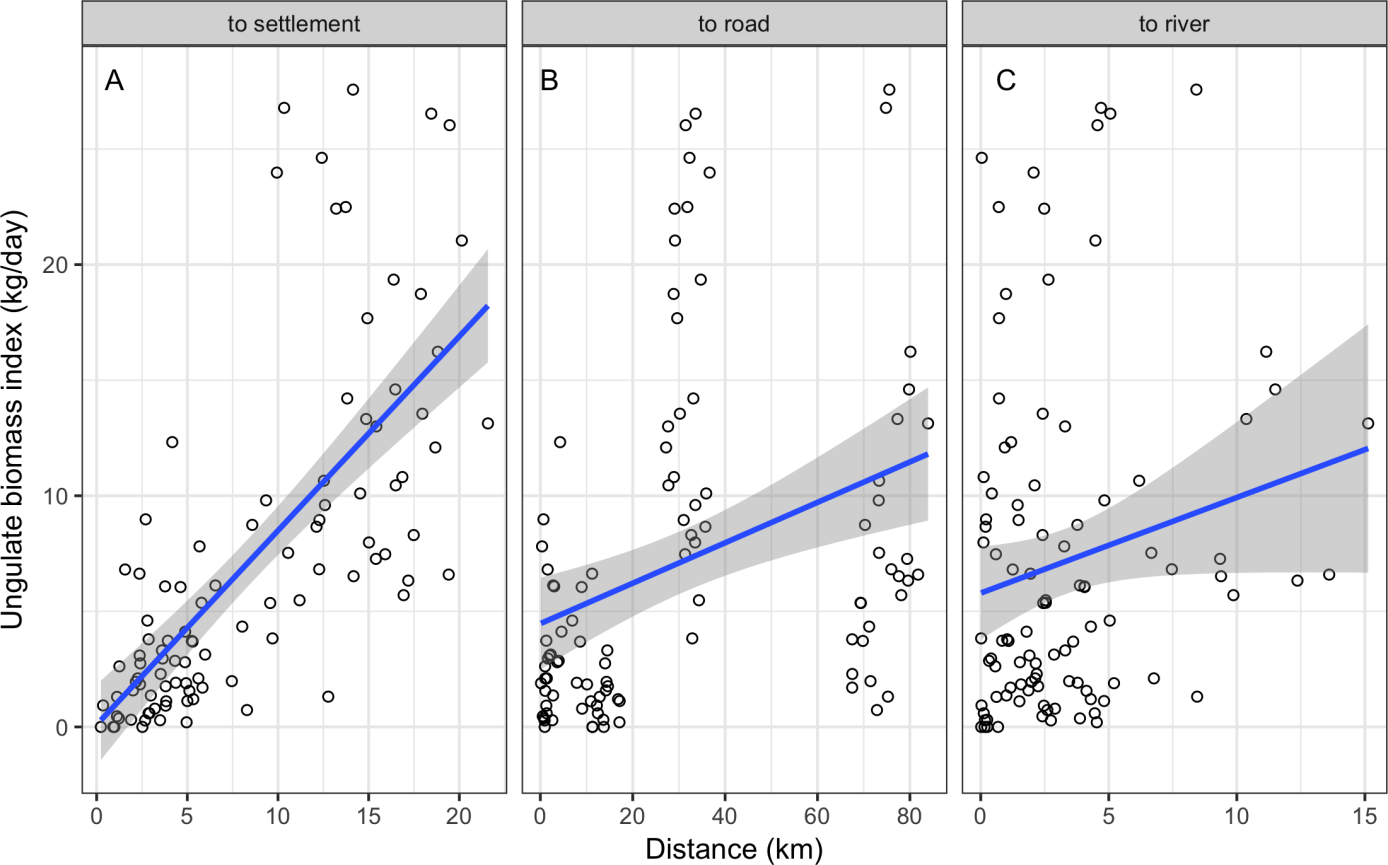
**Relationship between ungulate prey biomass and landscape accessibility measured as distance from camera trap stations (km) to settlements (A), roads (B) and rivers (C).** Data points are an index of ungulate biomass (kg/day) at each station.

We observed a positive relationship of ungulate prey biomass with distance to settlements (*r* = 0.71; *P* < 0.001; HDI_95_ = 0.61 to 0.79; Fig 3A) and distance to roads (*r* = 0.34; *P* < 0.001; HDI_95_ = 0.21 to 0.48; Fig 3B), and a marginally significant relationship with access to rivers (*r* = 0.19; *P* = 0.06, HDI_95_ = 0.02 to 0.33; Fig 3C).

### Jaguar abundance in Yasuní

**Fig 4:**
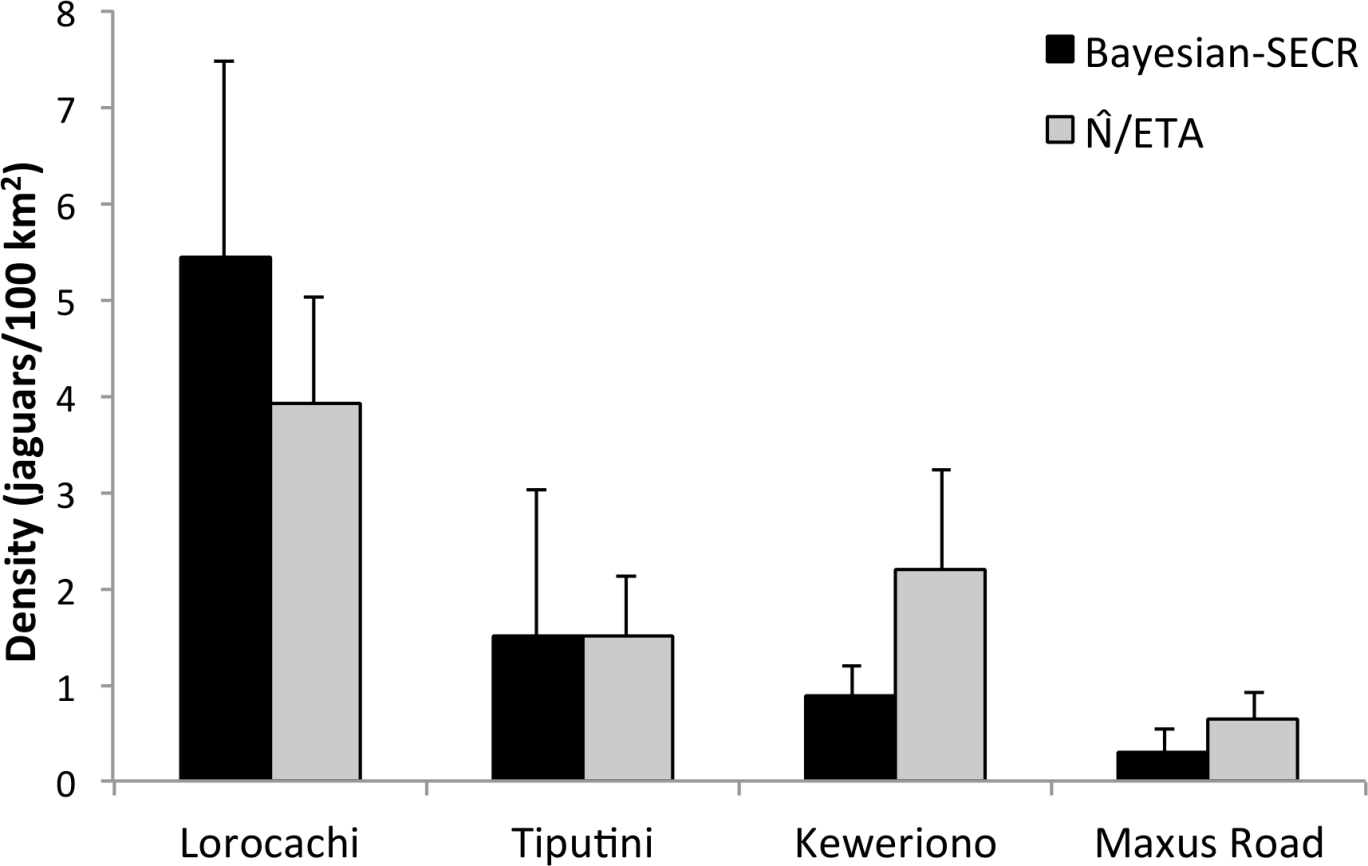
Jaguar density estimates at four sites in Yasuní Biosphere Reserve. Sites are arranged from least accessible to most accessible. Error bars correspond to SD and SE for Bayesian-SECR and $\hat{N}/ETA$ methods respectively.

Density estimates were based on a total of 59 captures that corresponded to 30 adult jaguars (18 males, 7 females and 5 un-sexed animals, S4 Table). A total of 13 jaguars were captured in Lorocachi (our most isolated study site), 6 in Tiputini, 8 in Keweriono and 3 in Maxus Road (our most accessible study site). Both, the spatially-explicit and the $\hat{N}/ETA$ methods provided similar results. For SECR analyses, we achieved convergence for *N*_*super*_ and *ψ* at all sites but not for *σ* and *λ*_*0*_ (S5 Table). Density decreased from 5.44 ± SD 2.04 individuals/100 km^2^ in our least accessible site to 0.29 ± SD 0.26 individuals/100 km^2^ in our most accessible site (Fig 4; S6 Table). For the $\hat{N}/ETA$ method we obtained effective trapping areas that varied from 458–486 km^2^ (S6 Table) and jaguar densities followed a similar pattern to SECR analyses, with a maximum density of 3.91 ± SE 1.11 and a minimum of 0.65 ± SE 0.26 individuals/100 km^2^ in our least and most accessible sites, respectively (Fig 4; S6 Table). The assumption of demographic closure for non-spatial models held for all four sites (S6 Table).

## Discussion

We show that facilitation of hunter access to a natural landscape can lead to development-induced impacts on predators and prey that end with reducing abundance of jaguar, the top predator of our study system. Our findings clearly support our first hypothesis that higher landscape accessibility (i.e., proximity to settlements, roads and rivers) to hunters leads to reduction of prey. These results are consistent with studies that show hunting effort is higher near settlements or sources of access [18,19,63]. Occurrence and biomass of the two species of peccaries, the species most harvested by Waorani [19,32–34], along with tapir and two species of brocket deer, were reduced in areas highly accessible to hunters. This pattern was evident even though our study sites were within a large relatively intact forest where source-sink dynamics could have diluted our capacity to detect such patterns [17].

Our second hypothesis, that reduction of large game from hunting would cause local declines in jaguar density, was framed based on the close relationship observed between abundance of large-bodied prey and other large cats [24,26]. Although jaguars are opportunistic predators, they are known to preferentially use large prey when available such as capybara (*Hydrochoerus hydrochaeris*), caiman (*Caiman crocodilus*), and especially white-lipped and collared peccaries [39]. Therefore, we expected high jaguar densities at sites where access to hunters is limited and ungulate populations are high. The relationship between jaguar abundance and human access followed our predictions, and estimates of jaguar density indicated that jaguar abundance in our least accessible site, Lorocachi, was 6–18 times higher (estimates from $\hat{N}/ETA$ and SECR, respectively) than in our most accessible site, Maxus Road. These results, coupled with the fact that prey occurrence and biomass were significantly lower in the most accessible sites, support our second hypothesis. Alternatively, if poaching of jaguars is higher in sites that are more accessible to hunters, consequences of reduction of the prey base for jaguars and direct hunting of jaguars could be confounded. We cannot completely rule out these confounding effects without more data on jaguar mortality; however, this would not change our most important conclusion, that prey and predator abundance decline with human access. Also, our study sites offered an advantage over many other areas in terms of minimizing the confounding of effects of prey decline and direct mortality on jaguars. The Waorani and Kichwa, primary inhabitants of the region, did not persecute jaguars systematically at the time of this study. For the Waorani, the jaguar is still a strong cultural symbol; they believe warriors become jaguars in an afterlife and that shamans become jaguars when they enter the forest to acquire powers for healing [64]. Hunting at the Maxus Road site was practiced only by Waorani who live in this area and in 14 months of hunting surveys along this site we only recorded one adult male killed and one kitten trapped by Waorani [19]. Also, we did not hear about jaguars killed in the other study sites during our 3 years of fieldwork. The lack of evidence for anthropogenic mortality of jaguars supports our conclusion that the negative relationship between jaguar density and human access likely results from reduction in prey when areas become accessible to hunters.

Human access is superimposed on a heterogeneous landscape where other factors also contribute to differences in fauna among sites. Prey biomass, especially white-lipped peccaries, was particularly high in Tiputini. Across Amazonia, white-lipped peccaries are strongly associated with wetlands dominated by the palm *Mauritia flexuosa* [65,66]. Palm wetlands are more common at Tiputini and the heavily hunted Maxus Road site than at our other sites (15.7% and 8.3% of the area of trap arrays, respectively, vs. < 1%; [67]). The abundant palm forests may explain the high biomass of white-lipped peccaries in Tiputini where human hunting was low. In contrast, harvesting of white-lipped peccaries by Waorani on Maxus Road is intense (>50% of total biomass of harvested bushmeat; [19]), and the occurrence and biomass of this species was low in our surveys even though highly favorable habitat was present.

Unexpectedly, although Tiputini had the highest occurrence and biomass of ungulates, this site did not have the highest jaguar density. Some plausible explanations include: 1) behavioral factors [68], for example, deterrence of other jaguars by a dominant male; 2) heterogeneity in detection probability [69] caused by differences in landscape composition across sites and not modeled because of limited data; and 3) edge effects associated with the location of this site at the reserve boundary [70]; during our research at Tiputini we observed commercial hunters entering our study area through the Tiputini River, and it is possible jaguars were poached for their parts in this area.

Across the range of jaguars, a key challenge for understanding threats to jaguar populations is the logistical difficulty of obtaining robust density estimates. We followed recent recommendations for analysis of camera trap data for jaguars [62], but our estimates of jaguar density should be used with caution (e.g., for comparison across studies) because, as with most studies, we were still limited by small samples sizes particularly at heavily hunted sites. As a result of the large home ranges of jaguars and low densities, sample designs that record large numbers of individuals and have low variance estimates are rare [62]. For example, to date, the jaguar survey with the largest spatial extent covered 1,320 km^2^, deployed 119 camera trap stations, and only captured 10 individuals [71], which are fewer individuals than we recorded at our least accessible site (13 jaguars).

If roads networks—and their associated colonization processes—continue to expand, the chances of conserving jaguars in Yasuní will be significantly reduced. Population viability assessments for jaguars indicate that a population of 650 individuals has a 97–100% probability of persisting for 200 years with minimal loss of heterozygosity [72]; this population number is similar to effective population sizes generally suggested for maintaining evolutionary potential in perpetuity [73,74]. If jaguar density across Yasuní Biosphere Reserve (c. 18,000 km^2^) is similar to our highest estimates (3.9–5.4 individuals/100 km^2^), this area could have high probability of sustaining a large enough population (i.e., 700–1,000 individuals) to meet these viability targets. However, maintaining this population requires addressing the problems of road expansion and associated hunting.

Jaguar conservation efforts to date include identification of key areas for jaguar conservation across the species range (Jaguar Conservation Units, JCUs [5]) and an ambitious initiative to connect these JCUs through a regional network of biological corridors [75]. Our results point to the need to coordinate these initiatives with local governments so that development activities such as road building are minimal near JCUs and jaguar corridors. In addition to landscape-scale conservation initiatives, we also believe significant effort needs to be invested in the management of jaguar prey at local levels. For example, populations of white-lipped peccaries, one of the most important prey for jaguar, are threatened throughout their range by hunting and habitat loss [76,77]. However, current information on management alternatives for peccaries and other prey is limited. We conducted a rapid search in Web of Science using the keywords “peccary/ies + management”, and obtained 64 citations. Most studies focused on ecology of peccaries and effects of hunting by traditional or indigenous groups. Only two research projects examined alternative management strategies for peccaries; one presenting the possibility of farming peccaries to avoid overhunting [78] and another on commercialization of certified peccary pelts through community-based wildlife conservation programs [79,80]. These results substantiate the need to report and assess current wildlife management efforts and develop new alternatives for game management in the region.

## Conclusions

In conclusion, worldwide, space is an important limiting factor for the conservation of large carnivores and, as natural habitats are reduced, large protected areas become even more central to their survival [4,14,15,70]. Road construction within and near protected areas leads to increased accessibility of hunters, overharvest of prey, and reduced potential of these lands to sustain viable populations of large predators. These development-induced ecological impacts also undoubtedly extend beyond population-level effects on jaguar and prey. Substantial evidence demonstrates that reduction in apex predators can alter composition, structure and functionality of entire ecosystems [1,2,81]. Moreover, large carnivores such as jaguar are important umbrella species whose conservation can contribute to the maintenance of co-occurring mammal species [82]. In order to guarantee functional ecosystems for future generations, where large carnivores are present, governments and funders of government initiatives should carefully evaluate placement of new development activities and infrastructure across the landscape, and exclude protected areas from these activities.

## Supporting information

S1 Appendix Selection (AIC) of occupancy models to explore occurrence of 8 prey species as a function of landscape access by hunters in Yasuní Biosphere Reserve. (PDF)

S1 Data Datasets for site occupancy estimation of jaguar prey species in Yasuní Biosphere Reserve. (XLSX)

S2 Data Datasets for density estimation of jaguar in Yasuní Biosphere Reserve. (XLSX)

S1 Table Survey effort with camera trap stations at four study sites in Yasuní Biosphere Reserve. (PDF)

S2 Table Settings for running SECR models with SPACECAP. (PDF)

S3 Table Biomass estimates (kg/100 trap-days) of potential prey species in Yasuní Biosphere Reserve. (PDF)

S4 Table Capture history of 30 jaguars used for abundance estimation in the four areas in Yasuní Biosphere Reserve. (PDF)

S5 Table Results of the Geweke diagnostic. (PDF)

S6 Table Jaguar density estimation with non-spatial and spatially-explicit models. (PDF)
