# Supplementary material for: When roads appear jaguars decline: Increased access to an Amazonian wilderness area reduces potential for jaguar conservation
Source: PLoS One. 2018 Jan 3;13(1):e0189740. doi: 10.1371/journal.pone.0189740 (PMC5751993; doi:10.1371/journal.pone.0189740)
Supplement: S1 Appendix — (PDF) [file pone.0189740.s001.pdf]

S1 Appendix. Selection (AIC) of occupancy models to explore occurrence of 8 prey species as a function of landscape access by hunters in Yasuní Biosphere Reserve. Models are ranked based on AIC_c (highest ranked model has lowest AIC_c). K = number of parameters estimated in model; ω = AIC_c weight; ω_+= support of predictor variable; Ψ = site occupancy probability; p = detection probability. Covariates include: settle = distance from camera to settlement (km); access = distance from camera to road or navigable river (km); habitat = topographic category (ridge or valley); DC = distance (m) between paired cameras.

White-lipped peccary *Tayassu pecari*

Model	AIC_c	ΔAIC_c	K	ω
Ψ(settle),p(DC)	481.88	0.00	4	0.18
Ψ(settle),p(.)	481.90	0.02	3	0.18
Ψ(settle+habitat),p(DC)	482.22	0.34	5	0.15
Ψ(access+settle+habitat),p(.)	482.60	0.72	5	0.13
Ψ(settle+habitat),p(.)	482.42	0.54	4	0.14
Ψ(access+settle),p(.)	482.88	1.00	4	0.11
Ψ(access+settle),p(DC)	483.80	1.92	5	0.07
Ψ(access+settle+habitat),p(DC)	485.28	3.40	6	0.03
Ψ(access),p(DC)	537.47	55.59	4	0.00
Ψ(access+habitat),p(DC)	538.67	56.79	5	0.00
Ψ(access),p(.)	539.48	57.60	3	0.00
Ψ(access+habitat),p(.)	540.59	58.71	4	0.00
Ψ(habitat),p(DC)	544.77	62.89	4	0.00
Ψ(.),p(.)	546.45	64.57	2	0.00
Ψ(habitat),p(.)	548.47	66.59	3	0.00

Predictor	ω_+
Access	0.34
Settlement	1.00
Habitat	0.46

Collared peccary *Pecari tajacu*

Model	AIC_c	ΔAIC_c	K	ω
Ψ(access+settle),p(DC)	975.30	0.00	5	0.33
Ψ(access+settle),p(.)	975.52	0.22	4	0.30
Ψ(access+settle+habitat),p(DC)	977.01	1.71	6	0.14
Ψ(access+settle+habitat),p(.)	977.21	1.91	5	0.13
Ψ(settle),p(DC)	979.34	4.04	4	0.04
Ψ(settle),p(.)	979.80	4.50	3	0.03
Ψ(settle+habitat),p(DC)	981.52	6.22	5	0.01
Ψ(settle+habitat),p(.)*	981.93	6.63	4	0.01
Ψ(access),p(DC)	989.66	14.36	4	0.00
Ψ(access),p(.)	990.19	14.89	3	0.00
Ψ(access+habitat),p(DC)	990.58	15.28	5	0.00
Ψ(access+habitat),p(.)	991.19	15.89	4	0.00
Ψ(.),p(.)	1006.30	31.01	2	0.00
Ψ(habitat),p(DC)	1007.37	32.07	4	0.00
Ψ(habitat),p(.)	1008.43	33.13	3	0.00

Predictor	ω_+
Access	0.89
Settlement	1.00
Habitat	0.29

* Model did not converge.

Amazonian tapir *Tapirus terrestris*

Model	AIC_c	ΔAIC_c	K	ω
Ψ(settle),p(DC)	630.52	0.00	4	0.50
Ψ(access+settle),p(DC)	632.36	1.84	5	0.20
Ψ(settle+habitat),p(DC)	632.72	2.20	5	0.17
Ψ(access+settle+habitat),p(DC)	634.62	4.10	6	0.06
Ψ(access+settle),p(.)	634.65	4.13	4	0.06
Ψ(access),p(DC)	642.82	12.30	4	0.00
Ψ(access+habitat),p(DC)	644.82	14.30	5	0.00
Ψ(habitat),p(DC)	645.04	14.52	4	0.00

Ψ(access),p(.)	649.09	18.57	3	0.00
Ψ(access+settle+habitat),p(.)*	650.64	20.12	5	0.00
Ψ(.),p(.)	650.49	19.97	2	0.00
Ψ(habitat),p(.)	652.62	22.10	3	0.00
Ψ(settle),p(.)	656.90	26.38	3	0.00
Ψ(settle+habitat),p(.)	659.07	28.55	4	0.00
Ψ(access+habitat),p(.)	660.05	29.53	4	0.00

Predictor	ω_+
Access	0.33
Settlement	1.00
Habitat	0.23

* Model did not converge.

Red brocket *Mazama americana*

Model	AIC_c	ΔAIC_c	K	ω
Ψ(settle),p(DC)	870.61	0.00	4	0.32
Ψ(settle+habitat),p(DC)	871.22	0.61	5	0.24
Ψ(access+settle),p(DC)	872.78	2.17	5	0.11
Ψ(access+settle+habitat),p(DC)	873.12	2.51	6	0.09
Ψ(access+habitat),p(DC)	873.42	2.81	5	0.08
Ψ(habitat),p(DC)	873.28	2.67	4	0.08
Ψ(access),p(DC)	873.59	2.98	4	0.07
Ψ(settle),p(.)	883.50	12.89	3	0.00
Ψ(settle+habitat),p(.)	884.08	13.47	4	0.00
Ψ(access+settle+habitat),p(.)	885.07	14.46	5	0.00
Ψ(access+settle),p(.)	885.45	14.84	4	0.00
Ψ(access+habitat),p(.)	887.32	16.71	4	0.00
Ψ(access),p(.)	888.19	17.58	3	0.00
Ψ(.),p(.)	888.73	18.12	2	0.00
Ψ(habitat),p(.)	889.52	18.91	3	0.00

Predictor	ω_+
Access	0.35
Settlement	0.76
Habitat	0.49

Amazonian brown brocket *Mazama nemorivaga*

Model	AIC_c	ΔAIC_c	K	ω
Ψ(settle+habitat),p(.)	332.21	0.00	4	0.27
Ψ(access+settle+habitat),p(.)	333.38	1.17	5	0.15
Ψ(access+settle),p(.)	333.51	1.30	4	0.14
Ψ(settle),p(.)	333.41	1.20	3	0.15
Ψ(settle+habitat),p(DC)	334.21	2.00	5	0.10
Ψ(access+settle+habitat),p(DC)	335.44	3.23	6	0.05
Ψ(access+settle),p(DC)	335.62	3.41	5	0.05
Ψ(settle),p(DC)	335.42	3.21	4	0.05
Ψ(habitat),p(.)	338.48	6.27	3	0.01
Ψ(.),p(.)	338.94	6.73	2	0.01
Ψ(habitat),p(DC)	339.65	7.44	4	0.01
Ψ(access+habitat),p(.)	340.18	7.97	4	0.00
Ψ(access),p(.)	340.97	8.76	3	0.00
Ψ(access+habitat),p(DC)	341.52	9.31	5	0.00
Ψ(access),p(DC)	342.46	10.25	4	0.00

Predictor	ω_+
Access	0.40
Settlement	0.96
Habitat	0.60

Paca *Cuniculus paca*

Model	AIC_c	ΔAIC_c	K	ω
Ψ(settle+habitat),p(.)	573.63	0.00	4	0.32
Ψ(access+settle+habitat),p(.)	574.21	0.58	5	0.24
Ψ(settle+habitat),p(DC)	575.66	2.03	5	0.11
Ψ(access+settle+habitat),p(DC)	576.30	2.67	6	0.08
Ψ(access+settle),p(.)	576.47	2.84	4	0.08

Ψ(settle),p(.)	576.96	3.33	3	0.06
Ψ(access+settle),p(DC)	578.49	4.86	5	0.03
Ψ(habitat),p(.)	578.64	5.01	3	0.03
Ψ(settle),p(DC)	578.94	5.31	4	0.02
Ψ(.),p(.)	580.47	6.84	2	0.01
Ψ(access+habitat),p(.)	580.77	7.14	4	0.01
Ψ(habitat),p(DC)	580.81	7.18	4	0.01
Ψ(access),p(.)	582.54	8.91	3	0.00
Ψ(access+habitat),p(DC)	582.99	9.36	5	0.00
Ψ(access),p(DC)	584.71	11.08	4	0.00
			**Predictor**	**ω_+**
			Access	0.44
			Settlement	0.94
			Habitat	0.80

Black agouti *Dasyprocta fuliginosa*

**Model**	**AIC_c**	**ΔAIC_c**	**K**	**ω**
Ψ(habitat),p(.)	1013.89	0.00	3	0.20
Ψ(.),p(.)	1013.98	0.09	2	0.19
Ψ(habitat),p(DC)	1015.28	1.39	4	0.10
Ψ(access+habitat),p(.)	1015.85	1.96	4	0.07
Ψ(settle+habitat),p(.)	1015.93	2.04	4	0.07
Ψ(settle),p(.)	1015.92	2.03	3	0.07
Ψ(access),p(.)	1016.09	2.20	3	0.07
Ψ(settle+habitat),p(DC)	1017.32	3.43	5	0.04
Ψ(access+habitat),p(DC)	1017.32	3.43	5	0.04
Ψ(settle),p(DC)	1017.21	3.32	4	0.04
Ψ(access+settle+habitat),p(.)	1017.54	3.65	5	0.03
Ψ(access),p(DC)	1017.46	3.57	4	0.03
Ψ(access+settle),p(.)	1017.92	4.03	4	0.03
Ψ(access+settle+habitat),p(DC)	1018.97	5.08	6	0.02
Ψ(access+settle),p(DC)	1019.26	5.37	5	0.01
			**Predictor**	**ω_+**
			Access	0.30

Settlement	0.30
Habitat	0.56

Armadillos (*Dasypus novemcinctus* and *D. kappleri*)

Model	AIC_c	ΔAIC_c	K	ω
Ψ(.),p(.)	664.82	0.00	2	0.26
Ψ(settle),p(DC)	665.57	0.75	4	0.18
Ψ(access+settle),p(DC)	666.05	1.22	5	0.14
Ψ(habitat),p(.)	666.76	1.94	3	0.10
Ψ(access),p(.)	666.87	2.05	3	0.09
Ψ(settle+habitat),p(DC)	667.64	2.81	5	0.06
Ψ(access+settle+habitat),p(DC)	668.29	3.47	6	0.05
Ψ(habitat),p(DC)	668.37	3.55	4	0.04
Ψ(access),p(DC)	668.49	3.67	4	0.04
Ψ(access+habitat),p(.)	668.89	4.07	4	0.03
Ψ(access+habitat),p(DC)	670.57	5.74	5	0.01
Ψ(settle+habitat),p(.)*	687.47	22.65	4	0.00
Ψ(access+settle),p(.)*	687.47	22.65	4	0.00
Ψ(settle),p(.)*	688.02	23.20	3	0.00
Ψ(access+settle+habitat),p(.)*	689.69	24.86	5	0.00

Predictor	ω_+
Access	0.36
Settlement	0.42
Habitat	0.30

* Model does not converge.
