# Supplementary material for: When roads appear jaguars decline: Increased access to an Amazonian wilderness area reduces potential for jaguar conservation
Source: PLoS One. 2018 Jan 3;13(1):e0189740. doi: 10.1371/journal.pone.0189740 (PMC5751993; doi:10.1371/journal.pone.0189740)
Supplement: S1 Table — (PDF) [file pone.0189740.s004.pdf]

**S1 Table. Survey effort with camera trap stations at four study sites in Yasuní Biosphere Reserve.**

| | **Lorocachi** | **Tiputini** | **Keweriono** | **Maxus Road** |
|---|---|---|---|---|
| Camera trap stations | 26 | 25 | 23 | 26 |
| Starting survey date | 07/23/2008 | 12/01/2008 | 08/15/2009 | 12/01/2007 |
| Ending survey date | 10/20/2008 | 03/10/2009 | 11/12/2009 | 02/28/2008 |
| ETD | 2,275 | 1,901 | 1,972 | 2,241 |
| Camera failure | 2.78% | 15.51%* | 4.73% | 4.23% |
| MCP ($km^2$) | 110 | 110 | 106 | 104 |

MCP = Minimum convex polygon of camera trap array; ETD = Effective trapping days in 90-day survey period; ***** To have comparable trapping effort at Tiputini despite higher camera failure, we retained cameras at this site for 10 additional days, which gave an effort of 2,136 ETD. However, no jaguars were captured in this additional period. Therefore, density estimates only included the first 90 days for all four sites.
