# Supplementary material for: When roads appear jaguars decline: Increased access to an Amazonian wilderness area reduces potential for jaguar conservation
Source: PLoS One. 2018 Jan 3;13(1):e0189740. doi: 10.1371/journal.pone.0189740 (PMC5751993; doi:10.1371/journal.pone.0189740)
Supplement: S2 Table — (PDF) [file pone.0189740.s005.pdf]

S2 Table. Settings for running SECR models with SPACECAP.

Site	Iterations	Burn-in	Data augmentation (Total*)
Lorocachi	300 000	150 000	260 (273)
Tiputini	600 000	300 000	240 (246)
Keweriono	600 000	300 000	320 (328)
Maxus Road	600 000	300 000	120 (123)

*Total of individuals: augmented individuals + observed individuals
