# Supplementary material for: When roads appear jaguars decline: Increased access to an Amazonian wilderness area reduces potential for jaguar conservation
Source: PLoS One. 2018 Jan 3;13(1):e0189740. doi: 10.1371/journal.pone.0189740 (PMC5751993; doi:10.1371/journal.pone.0189740)
Supplement: S3 Table — (PDF) [file pone.0189740.s006.pdf]

S3 Table. Biomass estimates (kg/100 trap-days) of potential prey species in Yasuní Biosphere Reserve.

	Lorocachi	Tiputini	Keweriono	Maxus Road
**Ungulates**				
Tayassu pecari	198.99	600.95	0.00	13.01
Pecari tajacu	195.24	158.99	66.73	87.80
Mazama americana	117.37	142.68	60.14	33.72
Mazama nemorivaga	10.78	28.72	0.00	9.99
Tapirus terrestris	310.61	527.99	59.68	186.18
Subtotal ungulates	**832.99**	**1459.34**	**186.55**	**330.71**
**Other mammals**				
Atelocynus microtis	3.74	2.43	8.24	3.00
Cabassous unicinctus	0.00	0.17	0.00	0.00
Cuniculus paca	9.69	16.48	9.52	11.27
Dasyprocta fuliginosa	31.96	29.78	24.57	22.64
Dasypus spp.	4.50	12.45	3.70	10.56
Didelphis marsupialis	0.83	4.33	1.38	1.31
Eira barbara	1.35	4.98	2.52	1.49
Hydrochoerus hydrochaeris	0.00	0.00	2.26	3.84
Leopardus pardalis	57.03	40.42	74.67	49.30
Leopardus wiedii	0.40	1.22	0.61	0.39
Myoprocta pratti	0.31	11.47	3.54	3.36
Myrmecophaga tridactyla	14.42	11.03	0.00	21.21
Nasua nasua	2.76	2.04	1.06	1.66
Priodontes maximus	21.49	23.61	26.69	21.08
Procyon cancrivorus	0.00	0.58	0.00	1.11
Puma yagouaroundi	1.12	1.43	1.29	0.55
Speothos venaticus	0.51	0.00	0.00	0.25
Sylvilagus brasiliensis	0.00	0.45	0.23	0.63
Tamandua tetradactyla	1.37	1.20	0.68	1.53
Subtotal other mammals	**151.50**	**164.05**	**160.96**	**155.19**
**Birds**				
Mitu salvini	19.54	10.63	4.32	1.92
Nothocrax urumutum	0.22	0.51	1.02	0.22
Penelope jacquacu	0.04	0.97	0.50	0.51

Pipile cumanensis	0.00	0.08	0.00	0.04
Psophia crepitans	13.26	13.77	15.13	15.92
Subtotal Birds	**33.07**	**25.97**	**20.97**	**18.60**
**Grand Total**	**1017.56**	**1649.36**	**368.48**	**504.50**
