# Supplementary material for: When roads appear jaguars decline: Increased access to an Amazonian wilderness area reduces potential for jaguar conservation
Source: PLoS One. 2018 Jan 3;13(1):e0189740. doi: 10.1371/journal.pone.0189740 (PMC5751993; doi:10.1371/journal.pone.0189740)
Supplement: S4 Table — (PDF) [file pone.0189740.s007.pdf]

S4 Table. Capture history of 30 jaguars used for abundance estimation in the four areas in Yasuní Biosphere Reserve. LR = Lorocachi, TI = Tiputini, KW = Keweriono, MR = Maxus Road, MDM = maximum distance moved, MMDM = mean MDM.

**Individual**	**Sex**	**Captures**	**MDM**
LR 1	Male	4	8990
LR 2	Male	1	7531
LR 3	Female	1	0
LR 4	Male	2	2529
LR 5	Male	4	4526
LR 6	Male	5	4464
LR 7	Undefined	1	0
LR 8	Male	2	0
LR 9	Male	4	4078
LR 10	Male	1	0
LR 11	Female	1	0
LR 12	Male	1	0
LR 13	Undefined	1	0
TI 1	Female	2	9458
TI 2	Male	2	8838
TI 3	Female	1	0
TI 4	Male	2	2685
TI 5	Undefined	1	0
TI 6	Undefined	1	0
KW 1	Male	3	8100
KW 2	Female	3	2847
KW 3	Male	2	0
KW 4	Male	3	11 715
KW 5	Undefined	1	0
KW 6	Male	1	0
KW 7	Female	1	0
KW 8	Male	1	0
MR 1	Female	2	6011
MR 2	Male	3	5055
MR 3	Male	2	4393
		**MMDM***	**6081**

*Individuals with MDM = 0 were not included in estimation of MMDM.
