# Supplementary material for: When roads appear jaguars decline: Increased access to an Amazonian wilderness area reduces potential for jaguar conservation
Source: PLoS One. 2018 Jan 3;13(1):e0189740. doi: 10.1371/journal.pone.0189740 (PMC5751993; doi:10.1371/journal.pone.0189740)
Supplement: S5 Table — (PDF) [file pone.0189740.s008.pdf]

S5 Table. Results of the Geweke diagnostic (z-statistic).

Site	σ	λ_0	ψ	N_{super}
Lorocachi	0.9788	0.1590	-0.7761	-0.6641
Tiputini	-2.2751	3.2588	0.6288	0.5431
Keweriono	-5.8354	0.7161	-0.2731	-0.5321
Maxus Road	-12.5034	0.7824	-1.4736	-1.3719

λ_0 is the detection probability when the distance between an animal's home range center and camera trap equals zero; σ represents the spatial scale at which detection probability decreases; ψ is the fraction of augmented individuals that actually represents the true population; N_{super} is the population size in the state-space *S*. Values of the z-statistic between -1.6 and 1.6 indicate model convergence
