# Supplementary material for: When roads appear jaguars decline: Increased access to an Amazonian wilderness area reduces potential for jaguar conservation
Source: PLoS One. 2018 Jan 3;13(1):e0189740. doi: 10.1371/journal.pone.0189740 (PMC5751993; doi:10.1371/journal.pone.0189740)
Supplement: S6 Table — (PDF) [file pone.0189740.s009.pdf]

S6 Table. Jaguar density estimation with non-spatial and spatially-explicit models.

	Site			
N̂/ETA	**Lorocachi**	**Tiputini**	**Keweriono**	**Maxus Road**
Capture probability, $\hat{p}$	0.05	0.04	0.05	0.08
Closure test, *P*	0.92	0.07	0.31	0.75
$\hat{N}$ ± SE	19 ± 5.3	7 ± 2.9	10 ± 4.7	3 ± 1.2
ETA (km^2)	486	467	458	463
$D_{\hat{N}/ETA}$ (n/100 km^2) ± SE	3.91 ± 1.11	1.50 ± 0.63	2.18 ± 1.04	0.65 ± 0.26
CV (%) of *D*	28.4	42.0	47.8	40.0
**Bayesian-SECR**				
S	1681	1663	1557	1671
M	260	240	320	120
N_{super}	92.04 ± 34.56	22.39 ± 23.39	12.45 ± 4.35	4.73 ± 4.12
D_{SECR} (n/100 km^2) ± SD	5.44 ± 2.04	1.49 ± 1.55	0.89 ± 0.31	0.29 ± 0.26
CV (%) of *D*	37.5	104.0	35.2	86.2

$\hat{N}$ = population size estimated with program CAPTURE (Model M_h); *ETA* = effective trapping area; *D* = density; *S* = state-space, including pixels with non-suitable habitat for jaguar; *M* = number of individuals used for data augmentation; N_{super} = estimated population in *S*; *CV* = coefficient of variation.
